# Four new species and seven new records of *Promalactis* Meyrick, 1908 (Lepidoptera, Oecophoridae) from Laos

**DOI:** 10.3897/zookeys.900.39569

**Published:** 2019-12-31

**Authors:** Sora Kim, Yang-Seop Bae, Seunghwan Lee

**Affiliations:** 1 Research Institute for Agriculture and Life Sciences, Seoul National University, Seoul, 08826, South Korea Seoul National University Seoul South Korea; 2 Bio-Resource and Environmental Center, Division of Life Science, Incheon National University, Incheon, 22012, South Korea Incheon National University Incheon South Korea

**Keywords:** First record of genus, fungivores and scavengers, PKK National Park, systematics

## Abstract

The genus *Promalactis* Meyrick, 1908 is recorded for the first time from Laos in mainland Southeast Asia and four new species are described: *P. crassa***sp. nov.**, *P. retusa***sp. nov.**, *P. senispina***sp. nov.**, and *P. uniclavata***sp. nov.** Additionally, seven species are newly reported from the country: *P. albisquama* Kim & Park, *P. apicisetifera* Du & Wang, *P. bitrigona* Kim & Park, *P. zolotuhini* Lvovsky, *P. parasuzukiella* Wang, *P. suzukiella* (Matsumura), and *P. spiraliola* Kim. Distributional data and diagnoses and/or descriptions for all species in Laos are provided, along with illustrations of adults and genitalia.

## Introduction

*Promalactis* Meyrick, 1908 is one of the largest genera of the family Oecophoridae (Lepidoptera, Gelechioidea), comprising 341 species (Kim et al. 2018; Wang and Liu 2019). They are highly diverse in China and the Indomalayan realm (Kim et al. 2017b). *Promalactis*, which includes fungivores and scavengers, is close to the genera *Harpella* Schrank, *Oecophora* Latreille, and *Schiffermuelleria* Hübner in the recent phylogeny of Kim et al. (2016), but it can be distinguished from them in having the forewing ground color brownish yellow to yellowish brown with distinct markings, such as white bands, patches, or fuscous suffusions (Kim et al. 2017c), and in having R_4_ and R_5_ stalked (Kim et al. 2014).

**Figure 1.**
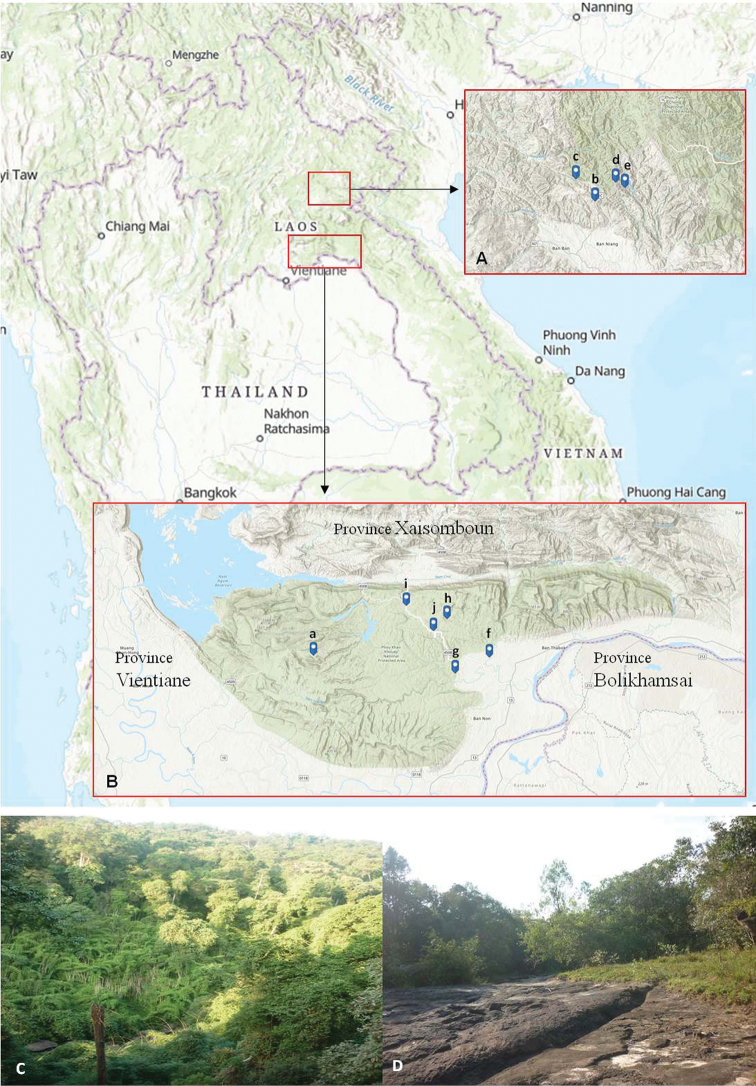
Locality of collection areas of *Promalactis* in Laos **A** Ban Tha area in Xiang khaung province **B** PKK National Park area **C** Forest of PKK National Park **D** collection sites near waterfall in PKK National Park.

In Mainland Southeast Asia (Fig. 1A), approximately 54 species of *Promalactis* have been reported: two species in Myanmar (Meyrick 1908b; Wang et al. 2013); six species in Thailand (Wang et al. 2013); 36 species in Vietnam (Lvovsky 1988, 1997, 2007, 2013; Kim et al. 2010, 2012, 2014) and 11 species in Cambodia (Kim et al. 2017c, 2018). However, no species has been recorded from Laos to date.

Laos is a andlocked and mountainous country located in Mainland Southeast Asia, bordered by Myanmar and China to the northwest, Vietnam to the east, Cambodia to the southwest, and Thailand to the west and southwest. It lies in the monsoon belt and experiences a rainy season between May and November, and a dry season from December to April.

The aim of this study is to report the first distributional data of *Promalactis* in Laos, with four new species described and seven other species newly recorded. Illustrations of all known species in Laos have been provided along with diagnosis and distributional information.

## Material and methods

Materials in this study were collected from 2012–2017 at several sites of three provinces of Laos, Vientiane (Northwest), Xiang khaung (Northeast), and Bolikhamsai (Central) (Fig. 1; Table 1). The collections were mainly from Phou Khao Khouay National Biodiversity Conservation Area (PKK National Park). It is considered as one of the most stunning preserves, with mixed deciduous forest dominating the lighter which less humid and shallow soils.

The forest consists of *Dipterocarpus* and *Shorea*, with a dry, evergreen forest centrally. Using either a mercury vapor lamp (220V/400W) or a black light lamp (20W), all individuals were taken alive, put in vials with cork lids, and killed with ammonia. Genitalia preparations for voucher specimens followed Kim et al. (2017a). All specimens were examined under a Leica 400B microscope (Leica Microsystems, Germany) and digital images were taken using Image Lab software, version 2.2.4.0 (MCM Design, Hillerød, Denmark). All specimens including type specimens will be deposited in two institutions: **SNU**, Seoul National University; **INU**, Incheon National University in Republic of Korea.

## Taxonomy

### Genus *Promalactis* Meyrick, 1908

*Promalactis* Meyrick, 1908a: 806. Type species. *Promalactis holozona* Meyrick, 1908a.

#### Promalactis parasuzukiella

Taxon classificationAnimaliaLepidopteraOecophoridae

Wang, 2006

DE729D36-FE25-5E85-B29A-BBACD9B9CDF8

**Figure 2.**
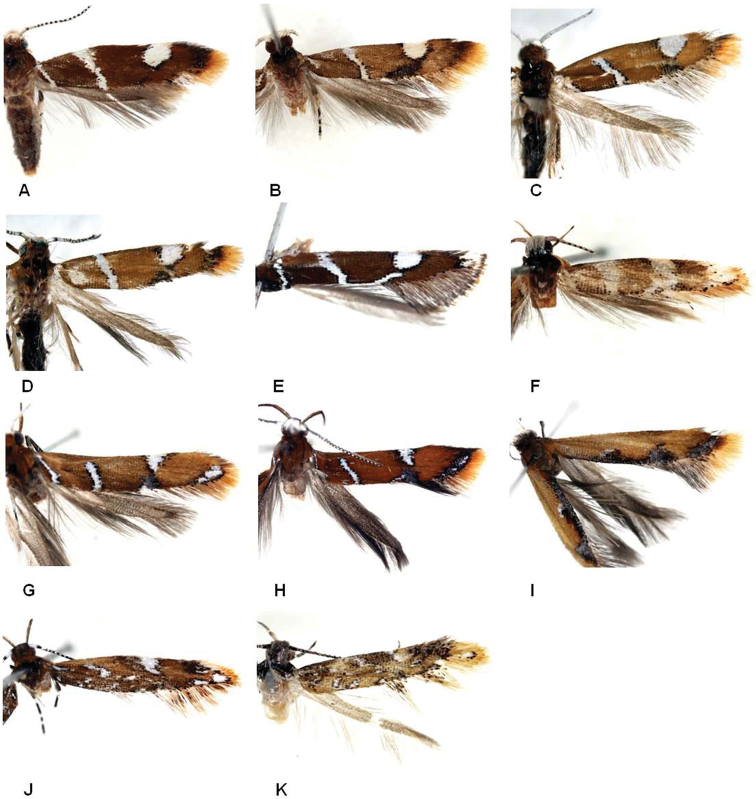
Wing pattern of Laos *Promalactis***A***Promalactis parasuzukiella***B***P. suzukiella***C***P. uniclavata* sp. nov. **D***P. albisquama***E***P. spiraliola***F***P. senispina* sp. nov. **G***P. apicisetifera***H***P. zolotuhini***I***P. bitrigona***J***P. crassa* sp. nov. **K***P. retusa* sp. nov.

**Figure 3.**
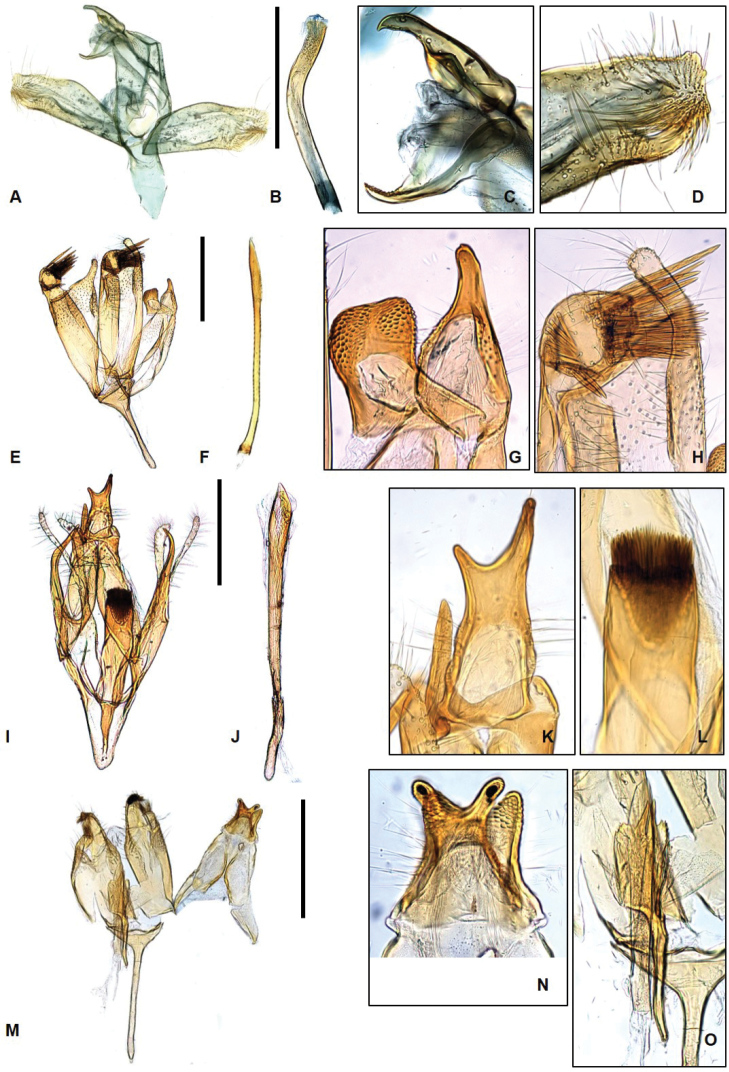
Genitalia of Laos *Promalactis***A–D***P. parasuzukiella*: **A** male genitalia **B** aedeagus **C** uncus and gnathos **D** apical part of valva **E–H***P. suzukiella*: **E** male genitalia **F** aedeagus **G** uncus and gnathos **H** apical part of valva **I–L***P. uniclavata* sp. nov.: **I** male genitalia **J** aedeagus **K** uncus and gnathos **L** dense hairs on apical part of juxta **M–O***P. albisquama*: **M** male genitalia **N** uncus and gnathos **O** juxta and aedeagus. Scale bars: 0.5 mm.

Figures 2A , 3A–D , 6A–C

Promalactis parasuzukiella Wang, 2006: 44. Type locality: China.

##### Material examined.

1♀, Laos, Bolikhamsai Prov., Hat Khay, 22 December 2012, Kim, gen. slide no. 9513/S. Kim; 1♀, Laos, Xiang khaung Prov., Ban Tha, 1298 m, 12 November 2015, Bae et al.; 1♂, Laos, Bolikhamsai prov., Phou Khao Khouay National Protected Area National Park, 322 m, 1 April 2016, Bae et al., gen. slide no. 9846/S. Kim; 1 extra (missing abdomen), Laos, Xiang khaung Prov., Ban Tha, 1298 m, 6 April 2016, Bae et al.; 1♀, same locality, 7 August 2017, Bae et al., gen. slide no. 9848/S. Kim.

##### Diagnosis.

This species (Fig. 2A) is externally most similar to *P. suzukiella* (Matsumura) in the forewing pattern, but it can be easily distinguishable from that species by the subbasal band which traverses from the costa to the posterior margin and the fuscous suffusion rarely developed under the circular costal patch at 2/3 of the forewing. The male genitalia (Fig. 3A–D) are differentiated from those of *P. suzukiella* in having the gnathos with a pointed apex and the aedeagus bent at 3/5 of its length. The female genitalia (Fig. 6A–C) are distinguished from those of *P. suzukiella* in having the ductus bursae bearing a long, serrated spine and another tiny spine.

##### Distribution.

Laos (northeast, central; new record), China (south) and Thailand (central).

**Table 1.** Collection sites and seasons of *Promalactis* in Laos.

**Province of Laos**	**Locality**	**Geographic position**	**Date, season**
Vientiane (Northwest)	PKK National Park	18°24'43.67”N, 102°50'57.92”E	7 December 2012, dry season
	Fig. 1B(a)		
Xiang khaung (Northeast)	Ban Tha, 1204 m	19°43'0.70”N, 103°35'24.20”E	30 June 2017, rainy season
	Fig. 1A(b)		7 August 2016, rainy season
	Ban Tha, 1298 m	19°45'07.35”N, 103°33'25.34”E	12 November 2015, rainy season
	Fig. 1A(c)		5–6 April 2016, dry season
			7 August 2017, rainy season
	Ban Tha, 1513 m	19°44'50.2”N, 103°37'28.1”E	20 February 2017, dry season
	Fig. 1A(d)		
	Ban Tha, 1524 m	19°44'17.99”N, 103°38'25.06”E	01 January 2017, dry season
	Fig. 1A(e)		
Bolikhamsai (Central)	Hat Khay	18°24'33.03”N, 103°9'37.08”E	22 December 2012, dry season
	Fig. 1B(f)		
	Thaphabath, Phaset	18°25'37.53”N, 103°16'19.64”E	21 February 2013, dry season
	Fig. 1B(g)		
	PKK National Park, 322 m	18°28'25.28”N, 103°5'6.11”E	01 April 2016, dry season
	Fig. 1B(h)		
	PKK National Park, 452 m	18°27'11.9”N, 103°03'40.5”E	03 July 2017, rainy season
	Fig. 1B(i)		
	PKK National Park, 561 m	18°29'43.79”N, 103°00'48.02”E	02 July 2017, rainy season
	Fig. 1B(j)		

#### Promalactis suzukiella

Taxon classificationAnimaliaLepidopteraOecophoridae

(Matsumura, 1931)

18DA9C51-30BB-524A-99B5-10B579BFC419

Figures 2B , 3E–H

Borkhausenia suzukiella Matsumura, 1931: 1089. Type locality: Japan.

##### Material examined.

1♂, Laos, Xiang khaung Prov., Ban Ta, 1524 m, 1 January 2017, Bae et al., gen. slide no. 9829/S. Kim.

##### Diagnosis.

This species (Fig. 2B) is similar to *P. uniclavata* sp. nov. in the wing pattern but can be easily distinguished from the latter in having the forewing without a subbasal band which traverses from the costa to the posterior margin, and in having the male genitalia (Fig. 3E–H) with the uncus having a thumb-like apex and a tougue-like gnathos.

##### Distribution.

Laos (northeast; new record), China (south), Vietnam; Palaearctic: Korea (South), China (northwest, northeast, central), Japan, Russia (Far East); Nearctic: USA.

#### Promalactis uniclavata

Taxon classificationAnimaliaLepidopteraOecophoridae

Kim sp. nov.

639C179F-C4DE-58A5-807F-E8D67E84ABD2

http://zoobank.org/32E673C6-160C-496B-A589-B2FD72AE5B95

Figures 2C , 3I–L , 6D–F

##### Type material.

**Holotype**: ♂, Laos, Xiang khaung Prov., Ban Tha, 1513 m, 20 February 2017, Bae et al., gen. slide no. 9834/S. Kim. Paratype: 1♂, 1♀, Laos, Vientiane Prov., Phou Khao Khouay National Protected Area, 7 December 2012, Lee et al., gen. slide no. 9512(M), 9509(F)/S. Kim; 1 ex., Laos, Bolikhamsai prov., Phou Khao Khouay National Protected Area, 322 m, 1 April 2016, Bae et al.; 1♂, Laos, Xiang khaung Prov., Ban Tha, 1298 m, 6 April 2016, Bae et al. [Holotype is deposited in INU, paratypes are separately deposited into SNU and INU.]

##### Diagnosis.

The species is superficially most similar to *P. suzukiella* in the similar forewing pattern but can be easily distinguished from the latter by the subbasal band which traverses from the costa to the posterior margin and with a fuscous suffusion below the costal patch at 2/3 of the posterior margin. The male genitalia are characterized in having the uncus bifurcate, the gnathos digitate, and the juxta large and club-shaped.

##### Description.

**Adult** (Fig. 2C). ***Head***: frons grayish brown tinged with white; vertex white, tinged with dark brown; occiput yellowish brown. ***Antenna***: scape white entirely, shorter than diameter of eye; flagellum white from base to 2/3 of its length, dorsally dark brown and white alternately from 2/3 to apex. ***Labial palpus***: 2^nd^ palpomere pale-yellow tinged with dark brown, as long as 3^rd^ palpomere; 3^rd^ palpomere dark brown, except pale yellowish white at apex dorsally. ***Thorax***: thorax and tegula dark brown. Wingspan 7.5–8.0 mm. Forewing ground color brownish yellow, darker near base; two bands and one costal patch all white, edged by fuscous scales: one subbasal band traversed from costal margin to posterior margin just near wing base; one antemedial band, oblique, not reaching costa; one costal patch semicircular at 3/4 of forewing, connected to fuscous suffusion before tornus; fuscous scales somewhat dense near apex; fringes grayish brown near tornus, yellow near apex. Hindwing ground color grayish brown; fringes grayish brown.

**Male genitalia** (Fig. 3I–L). Uncus wide at base, bifurcate distally, setose from base to middle; lateral lobes asymmetrical. Gnathos digitate, wide at base, shorter than uncus. Valva short; costal margin elongated, bifurcate, with different length of lobes, setose entirely; sacculus heavily sclerotized with pointed apex, shorter than costal margin. Juxta large, club-shaped, bearing U-shaped apical margin with dense hairs, as long as saccullus. Saccus large, triangular, longer then uncus. Aedeagus straight, gradually broader to apex, slightly longer than valva. Cornutus absent.

**Female genitalia** (Fig. 6D–F). Papillae anales setose. Apophyses posteriores almost twice longer than apophyses anteriores. Lamella postvaginalis large, incised medially, setose on caudal margin. Lamella antevaginalis wide, surrounded by dense hairs, concave on caudal margin. Antrum cylindrical, somewhat sclerotized posteriorly, gradually membranous anteriorly, as long as ductus bursae vertically. Ductus bursae membranous, sclerotized projection at 3/4 posteriorly. Corpus bursae small, circular; signum bearing one tiny spine and one sclerotized plate consisting of 3 or 4 tiny spines.

##### Distribution.

Laos (northeast, northwest, central; new).

##### Etymology.

The name is derived from the Latin, *uni* (= one) plus the Latin *clava* (= club), referring from the large, club-shaped juxta in the male genitalia.

#### Promalactis albisquama

Taxon classificationAnimaliaLepidopteraOecophoridae

Kim & Park, 2010

F0FD73E2-7A54-5847-9E70-1D390193F0DB

Figures 2D , 3M–O

Promalactis albisquama Kim & Park, 2010: 548. Type locality. Vietnam, Tamdao.

##### Material examined.

1♂, Laos, Vientiane Prov., Phou Khao Khouay National Protected Area, 7 December 2012, Lee et al., gen. slide no. 9511/S. Kim.

##### Diagnosis.

The species (Fig. 2D) is externally similar to *P. suzukiella* in the wing pattern but can be easily differentiated from the latter in having the forewing without a subbasal band and in having the fuscous suffusion denser near apex. The male genitalia (Fig. 3M–O) are also distinguished from those of *P. suzukiella* by the bifurcate uncus, the distinct juxta, and the extremely longer saccus.

##### Distribution.

Laos (northwest; new record), Cambodia (northwest), China (south), Vietnam (north).

#### Promalactis spiraliola

Taxon classificationAnimaliaLepidopteraOecophoridae

Kim, 2017

7BC8D1A3-DF7F-57DA-B07C-ED8919A83F51

**Figure 4.**
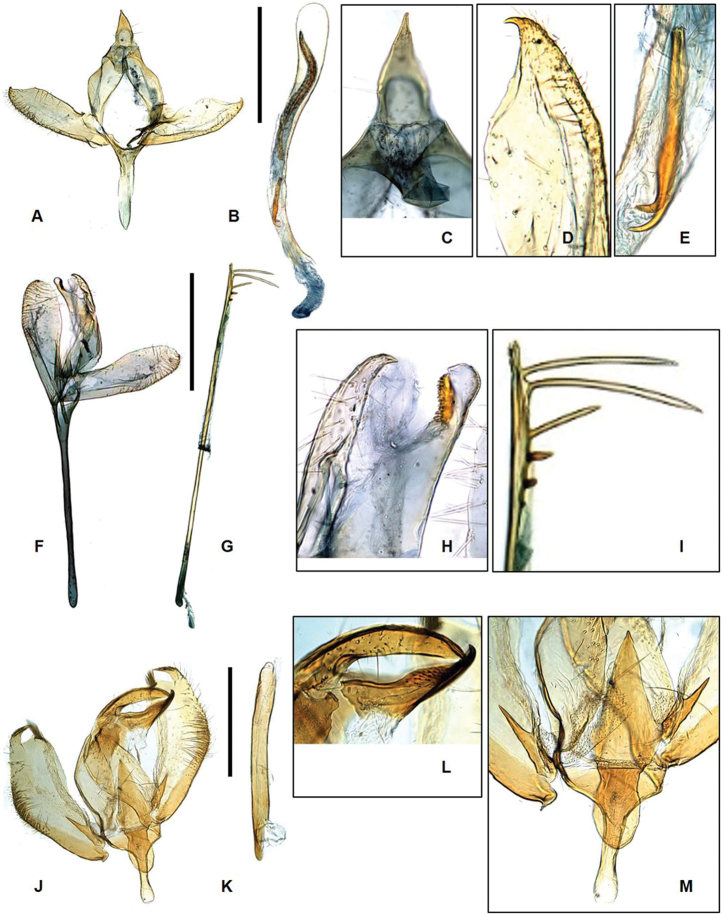
Genitalia of Laos *Promalactis***A–E***P. spiraliola*: **A** male genitalia **B** aedeagus **C** uncus and gnathos **D** apical part of valva **E** bifurcate part of cornutus **F–I***P. senispina* sp. nov.: **F** male genitalia **G** aedeagus **H** sclerotized projection of gnathos **I** apical projections of aedeagus **J–M***P. apicisetifera*: **J** male genitalia **K** aedeagus **L** uncus and gnathos **M** juxta. Scale bars: 0.5mm.

Figures 2E , 4A–E , 6G, H

Promalactis spiraliola Kim, 2017: 1710. Type locality: Cambodia, Koh Kong.

##### Material examined.

2♂, 1♀ and 1 ex., Bolikhamsai Prov., Phou Khao Khouay National Protected Area National Park, 561 m, 2 July 2017, gen. slide no. 9862(♂), 9858(♂) and 9863 (♀)/S. Kim. 1♂, Laos, Bolikhamsai prov., Phou Khao Khouay National Protected Area National Park, 561 m, 2 July 2017, Bae et al., gen. slide no. 9858/S. Kim.

##### Diagnosis.

The species (Fig. 2E) is externally characterized by the narrow, medial band not connected to the white costal patch on the forewing. The male genitalia (Fig. 4A–E) are also distinguished by the weakly developed gnathos and large aedeagus. The female genitalia (Fig. 6G, H) are characterized in having the ductus bursae coiled several times and bearing numerous tiny spines, and in having the corpus bursae small, membranous, and without signum.

##### Distribution.

Laos (central; new record), Cambodia (southwest).

#### Promalactis senispina

Taxon classificationAnimaliaLepidopteraOecophoridae

Kim sp. nov.

CEB01EE3-50F5-54C0-A0D2-E5FAB1A0F72D

http://zoobank.org/9468C7C7-C403-4D0A-BA93-E5D8D98DB353

Figures 2F , 4F–I

##### Type material.

**Holotype**: ♂, Laos, Bolikhamsai prov., Phou Khao Khouay National Protected Area National Park, 322 m, 1 April 2016, Bae et al., gen. slide no. 9860/S. Kim. [Holotype is deposited in INU.]

##### Diagnosis.

This species is externally similar to *P. lophacantha* Wang, Du & Li by having irregular bands of forewing, but it can be easily differentiated by the extended saccus and the prolonged aedeagus, which bears six projections in male genitalia.

##### Description.

**Adult** (Fig. 2F). ***Head***: frons pale yellowish white; vertex white; occiput white. Antenna: scape white entirely, longer than diameter of eye; flagellum dark brown and white alternately from base to apex dorsally. Labial palpus: 2^nd^ palpomere pale yellowish brown, slightly longer than 3^rd^ palpomere; 3^rd^ palpomere pale yellow tinged with white, except dark brown apically. ***Thorax***: thorax and tegula dark brown. Wingspan 7.0–7.5 mm. Forewing ground color yellowish brown; three bands, one costal patch, and one apical patch all white: one subbasal band traversed from costal margin to posterior margin near wing base; one antemedial band broad, slightly oblique toward wing base, traversed from costa to posterior margin; one medial band connected to postmedial short band on lower margin of cell; one apical patch accupied after 3/4 of forewing to apex; fuscous scales scattered followed by posterior margin of forewing; fringes yellow, except pale yellowish white near medial and postmedial bands. Hindwing ground color grayish dark brown; fringes dark grayish brown.

**Male genitalia** (Fig. 4F–I). Uncus wide at base, gradually narrowed to pointed apex, setose apically, as long as gnathos. Gnathos wide at base, gradually narrowed to apex, thumb-shaped apex, bearing sclerotized and serrated projection inner margin, as long as uncus. Valva symmetrical; costal margin convex after middle; saccular margin moderate, setose from 1/2 of its length to apex. Saccus extremely long, 1.5 times longer than valva. Aedeagus straight, elongated, as long as total length of genitalia, with six spines, differently sized apically; cornutus absent.

**Female** unknown.

##### Distribution.

Laos (central; new).

##### Etymology.

The specific name is derived from the Latin, *seni* (= six) and -*spina* (= spine), referring from the aedeagus bearing six spines in males.

#### Promalactis apicisetifera

Taxon classificationAnimaliaLepidopteraOecophoridae

Du, Li & Wang, 2011

7FC648F6-034A-5542-9B41-E8DB24D60C6C

Figures 2G , 4J–M

Promalactis apicesetifera Du, Li & Wang, 2011: 52. Type locality: China

##### Material examined.

1♂, Laos, Xiang khaung Prov., Ban Tha, 1298 m, 7 August 2017, Bae et al., gen. slide no. 9842/S. Kim.

##### Diagnosis.

The species (Fig. 2G) is similar to *P. zolotuhini* Lvovsky, 2013 in the wing pattern but can be easily recognized from the latter species by the yellowish-brown ground color and relatively broad subbasal band and the absence of medial band of the forewing. The male genitalia (Fig. 4J–M) are characterized in having the triangular juxta bearing lateral cylindrical lobes.

##### Distribution.

Laos (northeast; new record), China (south).

#### Promalactis zolotuhini

Taxon classificationAnimaliaLepidopteraOecophoridae

Lvovsky, 2013

25354FA5-C268-55CB-A520-896B61564C43

Figures 2H , 5A–D

Promalactis zolotuhoni Lvovsky, 2013: 133. Type locality: Vietnam.

##### Material examined.

1♂, Laos, Bolikhamsai Prov., Phou Khao Khouay National Protected, 452 m, 3 July 2017, Bae et al., gen. slide no. 9840/ S. Kim.

##### Diagnosis.

**Figure 5.**
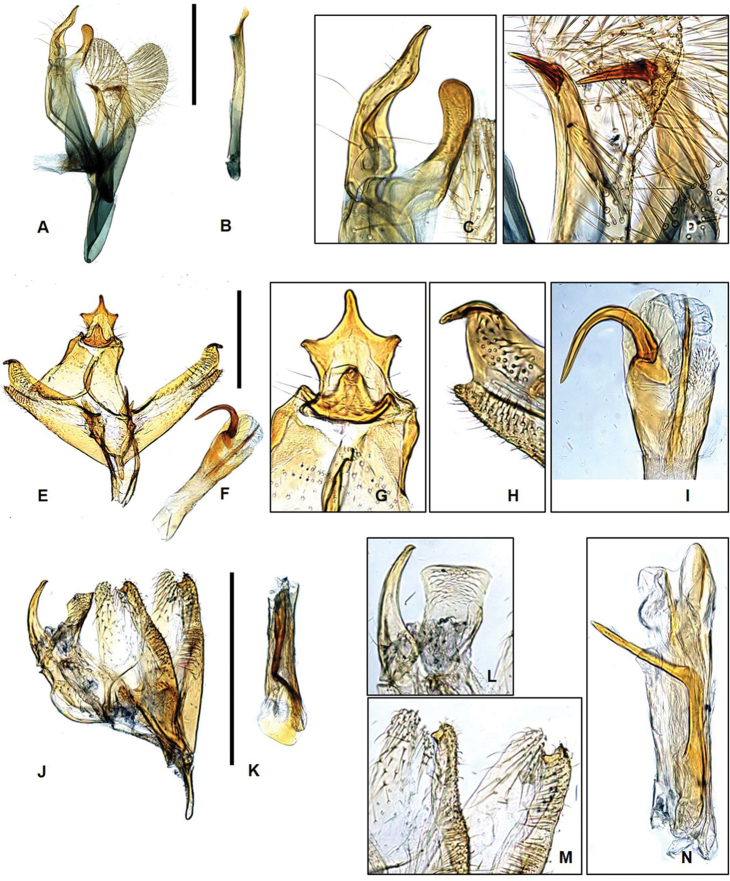
Genitalia of Laos *Promalactis***A–D***P. zolotuhini*: **A** male genitalia **B** aedeagus **C** uncus and gnathos **D** cucullus **E–I***P. crassa* sp. nov.: **E** male genitalia **F** aedeagus **G** uncus and gnathos **H** apical part of valva **I** cornutus and spine of aedeagus **J–N***P. retusa* sp. nov.: **J** male genitalia **K** aedeagus **L** uncus and gnathos **M** apical part of valva **N** cornutus. Scale bar: 0.5 mm.

This species (Fig. 2H) is close to *P. apicisetifera* Du & Wang in its wing pattern, but it can be easily distinguished in having the forewing ground color tinged with reddish dark brown. The male genitalia (Fig. 5A–D) are differentiated from the those of *P. apicisetifera* in having the gnathos bearing round apical tips, the cucullus sclerotized, and the valva rounded apically.

##### Distribution.

Laos (northeast; new record), Vietnam (north).

#### Promalactis bitrigona

Taxon classificationAnimaliaLepidopteraOecophoridae

Kim& Park, 2012

8EEF1C22-155B-5E8C-A84B-4CE56471AE42

Figures 2I , 7A–C

Promalactis bitrigona Kim & Park, 2012: 900. Type locality: Vietnam.

##### Material examined.

One female, Laos, Xiang khaung Prov., Ban Tha, 1204 m, 30 June 2017, Bae et al, gen. slide no. 9847/ S Kim.

##### Diagnosis.

The species (Fig. 2I) is distinguished from congeners by the three suffusions on the antemedial, postmedial, and apical areas of posterior margin of forewing. The male genitalia are characterized in having the valva with dense hairs on the costal margin (Kim et al. 2012; Fig. 3D–F), and by the female genitalia (Fig. 7A–C), which is characterized in having the antrum with a thumb-shaped caudal margin and the signum somewhat triangular-pyramid-shaped.

##### Distribution.

Laos (northeast; new record), Vietnam (north).

#### Promalactis crassa

Taxon classificationAnimaliaLepidopteraOecophoridae

Kim sp. nov.

692A2E27-ED9E-5470-9D5D-5EC79FBE1B80

http://zoobank.org/A61E6ECE-9D3B-483E-8CB6-C4D83A1294D3

Figures 2J , 5E–I , 7D–F

##### Type material.

**Holotype**: ♂, Laos, Xiang khaung Prov., Ban Tha, 1298 m, 7 August 2017, Bae et al., gen. slide no. 9592/ S. Kim. Paratype: 1♀, same locality, date and collector, gen. slide no. 9595. [Holotype and paratype are deposited in INU].

##### Diagnosis.

This species is superficially similar to *P. diorbis* Kim & Park, 2012, but it can be differentiated in having the large costal patch at 4/5 of the length of the forewing and in having the small antrum and the thick ductus bursae bearing several tiny spines in the female genitalia.

##### Description.

**Adult** (Fig. 2J). ***Head***: frons pale grayish dark brown, tinged with dark brown; vertex dark brown; occiput yellowish dark brown. Antenna: scape entirely white, except dark brown apically, shorter than diameter of eye; fragellum dark brown and white alternately from base to apex dorsally. ***Labial palpus***: 2^nd^ palpomere pale yellowish dark brown, 1.5 times longer than 3^rd^ palpomere; 2^nd^ palpomere dark brown, except white at apex. ***Thorax***: thorax blackish dark brown; tegula dark brown. Wing expanse 11.0–11.5 mm. Forewing ground color yellowish brown; five bands, one spot, two costal patches, one posterior patch, and one apical patch, all white edged with fuscous scales: one subbasal band short and one tiny spot near base, not connected each other; two antemedial bands: one just below subcostal (Sc) vein and the other at 1/6 posterior margin, both irregularly shaped, connected to each other at antemedial part of medial cell; the other antemedial band just before middle narrowed, arched connected to postmedial band; two costal patches: large one at 3/5 and somewhat semi-ovate, after pale grayish suffusion; small one at 4/5 and irregularly shaped, after fuscous suffusion; one small posterior parch after tornus; one apical patch larger than near costal and posterior patches; fringes yellowish brown near apex, mixed with fuscous scales near tornus. Hindwing more or less lanceolate; Hindwing ground color grayish brown; fringes dark grayish dark brown.

**Male genitalia** (Fig. 5E–I). Uncus large, thumb-shaped, with sclerotized, trifurcate projection, laterally setose near base. Gnathos wide at base, gradually narrowing to apex, inverted bell-shaped, shorter than uncus. Tegumen simple. Valva symmetrical; costal margin slightly concave basally, slightly convex medially, upward at sub-apex, bearing sclerotized, tiny spine at apex, setose after 2/3 to apex; saccular margin moderate, gradually narrowed to blunt apex, setose after 3/4 to apex, shorter than costal margin of valve. Juxta small bearing lateral spinous lobes, length of 2/3 of valve. Sacccus wide at base, triangular, longer than uncus. Aedeagus gradually narrowed from base to 1/5, straight from 1/5 to 1/2, gradually broader from 1/2 to apex, bearing spine with bifurcate apexes at middle, 1/2 length of aedeagus; cornutus large, heavily sclerotized, hook-shaped at sub-apex.

**Female genitalia** (Fig. 7D–F). Apophyses posteriors almost 1.5 times longer than apophyses anteriores. Apophyses anteriores as long as papillae anales. Lamella postveginalis small, bearing lateral circular lobes on causdal margin. Lamella antevaginalis wide at base, incised centrally. Antrum small, tiny cup-shaped. Ductus bursae thickly developed, wide at base, gradually narrowed from 4/5 to 3/5, somewhat straight from 3/5 to corpus bursae, wrinkled longitudinally after middle, with numerous scattered spines. Corpus bursae membranous, semi-ovate. Signum absent.

##### Distribution.

Laos (northeast; new).

##### Etymology.

The name of this species is derived from the Latin, *crass* (= thick) and the suffix –*a*, referring from the ductus bursae thickly developed in the female genitalia.

#### Promalactis retusa

Taxon classificationAnimaliaLepidopteraOecophoridae

Kim sp. nov.

B06BD01A-E67E-5C22-9E29-835815FF3613

http://zoobank.org/9D115097-1D7D-4C8F-B91E-51487818BB8A

Figures 2K , 5J–N

##### Type material.

**Holotype**: ♂, Laos, Bolikhamsai prov., Thaphabath, Phaset, 21 February 2013, Kim et al., gen. slide no. 9508/ S. Kim. Paratype: 1 ♂, same locality, data as holotype and collector. [Holotype and paratype are deposited in SNU.]

##### Diagnosis.

This species is externally similar to *P. crassa* sp. nov. in its wing pattern, but it can be easily recognized in having the costal and apical patches not large in the forewing. The male genitalia are differentiated by the inverted funnel-shaped uncus and the rectangular gnathos with blunt apex.

##### Description.

**Adult** (Fig. 2K). ***Head***: frons and vertex grayish dark brown; occiput grayish dark brown. Antenna: scape dark brown dorsally, white ventrally, shorter than diameter of eye; fragellum dark brown dorsally. ***Labial palpus***: 2^nd^ palpomere pale yellowish brown, except dark brown at apex dorsally, 1.5 times longer than 3^rd^ palpomere; 3^rd^ palpomere dark brown, except white at base dorsally. ***Thorax***: thorax grayish dark brown partly mixed with whitish scales; tegula dark grayish brown. Wing expanse 8.0–8.5mm. Forewing ground color yellowish brown tinged with grayish dark brown near wing base, middle, and sub-apex on costa; five bands, one costal patch, four spots, and one apical patch, all white edged with fuscous scales: two subbasal bands, one from Sc vein, oblique toward apex, the other from anal vein, oblique toward costa, both connected to each other; two antemedial bands at 1/5, 2/7 of posterior margin, short, oblique toward costa; one antemedial band at 1/3 of costa, oblique toward before tornus; two spots medially, one on posterior vein of medial cell, the other at 1/2 of posterior margin; one costal patch, not connected to under tiny spot on posterior vein of medial cell; one spot at tornus; one apical patch occupied after 6/7 to apex with fuscous scales; fringes yellow near apex, mixed with fuscous scales middle and near tornus. Hindwing more lanceolate; ground color pale grayish dark brown; fringes grayish brown.

**Male genitalia** (Fig. 5J–N) Uncus inverted funnel-shaped, wide at base, gradually narrowing to apex, slightly bent inward, longer than gnathos. Gnathos rectangular, concave laterally, blunt apically, gradually narrowed from base to 1/2, gradually broadened from 1/2 to apex. Tegumen simple, as long as uncus. Valva symmetrical; costal margin straight to apex, roundly edged, setose after middle; saccular margin moderate, gradually narrowed to apex, apex bearing tiny spine, setose after middle. Saccus short, finger-shaped, shorter than uncus. Aedesgus gradually broader to apex, as long as valve; cornutus sclerotized, bifurcate after middle.

**Female** unknown.

##### Distribution.

Laos (northeast; new).

##### Etymology.

The name of species is derived from the Latin, *retus* (= blunt) and the suffix –a, referring from the gnathos with blunt apex of male genitalia.

**Figure 6.**
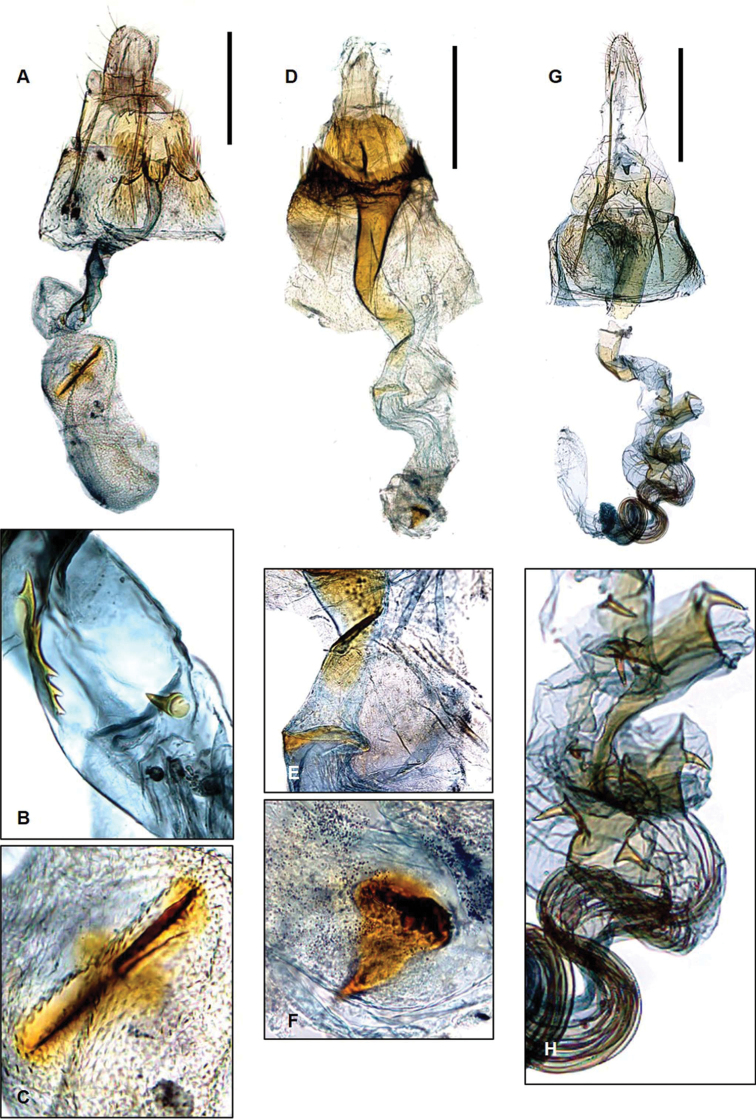
Genitalia of Laos *Promalactis***A–C***P. parasuzukiella*: **A** female genitalia **B** spines in ductus bursae **C** signum **D–F***P. uniclavata* sp. nov.: **D** female genitalia **E** projection in ductus bursae **F** signum **G–H***P. spiraliola*: **G** female genitalia **H** numerous spines in ductus bursae. Scale bars: 0.5 mm.

**Figure 7.**
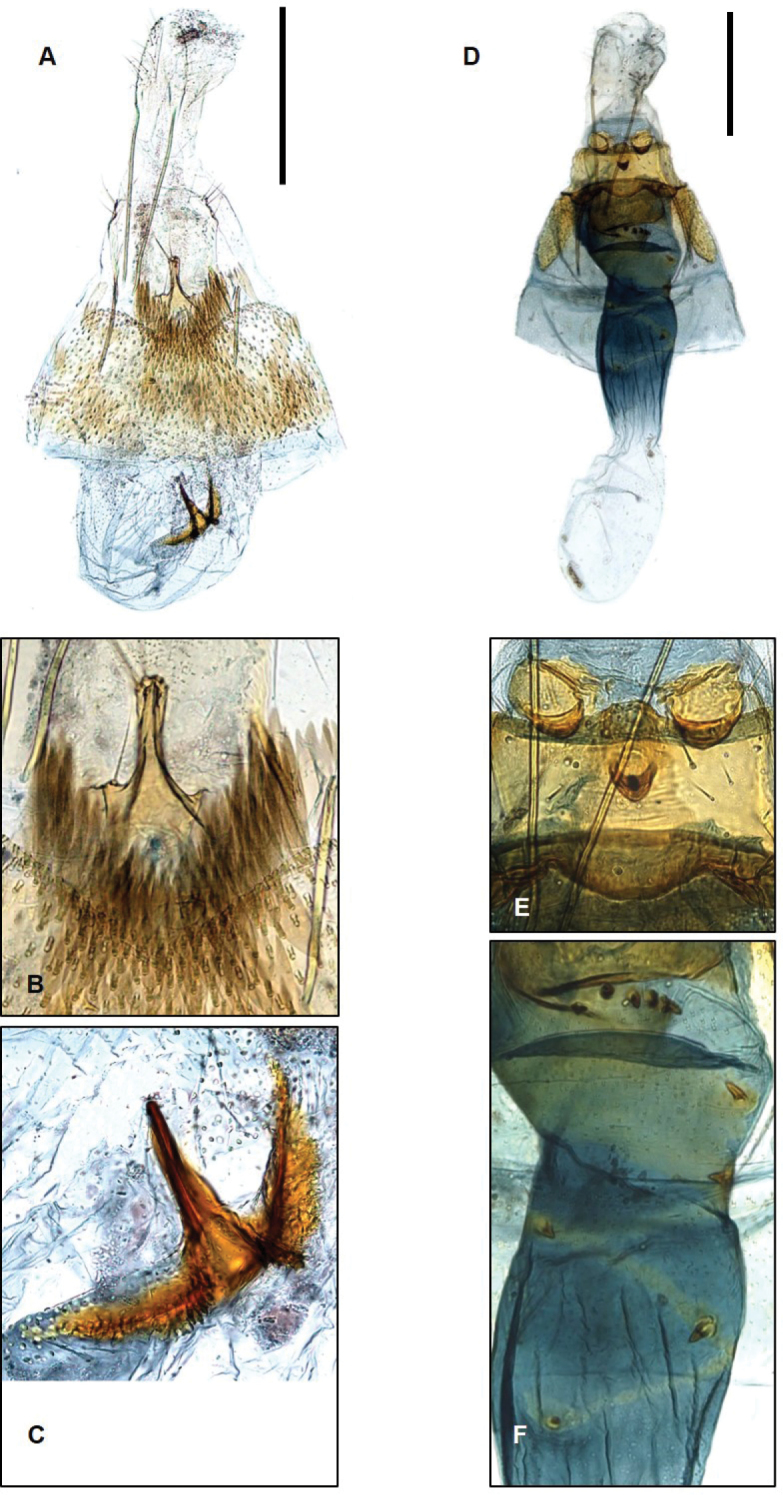
Genitalia of Laos *Promalactis***A–C***P. bitrigona*: **A** female genitalia **B** antrum **C** signum **D–F***P. crassa* sp. nov.: **D** female genitalia **E** lamella post-and ante-vaginalis **F** tiny spines in ductus bursae. Scale bars: 0.5 mm.

## Supplementary Material

XML Treatment for Promalactis parasuzukiella

XML Treatment for Promalactis suzukiella

XML Treatment for Promalactis uniclavata

XML Treatment for Promalactis albisquama

XML Treatment for Promalactis spiraliola

XML Treatment for Promalactis senispina

XML Treatment for Promalactis apicisetifera

XML Treatment for Promalactis zolotuhini

XML Treatment for Promalactis bitrigona

XML Treatment for Promalactis crassa

XML Treatment for Promalactis retusa
